# Collodion in Mammary Inflammations

**Published:** 1850-09

**Authors:** John Evans

**Affiliations:** Prof. of Obstetrics, etc., in Rush Medical College


### ARTICLE V.

*Collodion in Mammary Inflammation.*—By Jno. Evans, M. D., Prof. of Obstetrics, etc., in Rush Medical College.

Mammary Inflammation, whether it occurs during lactation or at any other time, is exceedingly prone to result in suppuration. This is owing to the great vascularity of the organ, and its loose and flaccid texture, which allows the determination of blood to it speedily to result in effusion of lymph, which, in the absence of the pressure made by denser structures, is not absorbed, but results in the secretion of pus.—Thus when the breast becomes indurated under the treatment heretofore practised by the professsion, of poulticing, applying liniments, tobacco leaves, &c., without constitutional treatment, it almost uniformly forms an abscess.

Disheartened by the general want of success in preventing suppuration by the ordinary means of treatment, and satisfied that the most prominent indication of cure was to overcome the freedom with which the blood is forced into the mamma, and, by compression, cause the absorption of the lymph, as is done by the roller applied on the extremities, in various forms of inflammation, I determined to use a complete coating of the collodion to obtain the benefit of its contraction; the result of which will be more fully illustrated by relating the following cases in which it has been used.

Case I.—Dec. 18th, 1849; called to see Mrs. J., suffering from Mammary Inflammation during lactation. Found she had bathed with liniments, applied poultices and kept the milk well drawn from the breast, but without apparent benefit. I could detect decided flucuation at the point of greatest suffering. I ordered a coat of the liquid adhesive plaster which gave very prompt relief, and the inflammation speedily subsided. A few days after I opened the abscess which pointed, but it discharged a very small quantity and rapidly recovered.

Case II.—Mrs. S. was confined June 5, 1850, with her third child. On the 7th, she was attacked with a chill, followed by high Fever and active inflammation of both mammæ. She had suffered, after each of her previous confinements, with extensive abscess of the left breast. The secretion of milk was very profuse, and notwithstanding strenuous efforts to keep the milk drawn off by the assistance of a little girl who drew it very freely, the left mamma became extensively indurated and was acutely sensitive and painful at the point of the former abscesses.

I applied the collodion so as to cover the indurations completely, with the effect of promptly relieving the suffering of the patient; and by repeating the coating morning and night for a few days the indurations were removed. The only additional treatment used was Seidlitz Powders given to produce a laxative effect.

Case III.—Mr. McC. eight days after confinement, was attacked with mammary inflamation attended with chill and high febrile excitement, on the 8th of June 1850.

Gave Mass Hyd. grs. xij with Pulv. Doveri grs. viij. in the evening.

June 9th. This morning the breast is very painful and extremely tender to the touch, great thirst, dry mouth, frequent but compressible pulse and a troublesome cough. The bowels had not been moved. Ordered Sul. Mag. ℥ss. and an application of the collodion to the breast, and free abstraction of milk.

June 10th. Much better, says the application of collodion to the inflamed breast gave immediate relief to the pain, and the soreness has rapidly diminished. Ordered the application to be repeated.

The recovery was rapid without suppuration.

Case IV.—Mrs. P. three weeks after confinement was attacked with inflammation of the mamma on the 24th of June 1850. She had been previously under treatment for uterine phlebitis, from which she was recovering.

The breast was swollen, indurated and very painful. The collodion was applied so as to cover the induration and swelling, with almost instant relief to the pain. By repeating the application the swelling subsided gradually, without suppuration.

Case V.—July 15th 1850. Mrs. M. had an attack of inflammation of the right mamma, about ten days after confinement. I was called the next day and found it swollen, indurated, and painful, notwithstanding the milk had been kept freely drawn and the breast well fomented. A coating with collodion as in the preceding cases, promptly gave relief to the pain, and gradually removed the swelling.

In no case where I have used the collodion, except the first one reported, has the slightest suppuration taken place.

In every instance the relief from suffering has been prompt, and no inconvenience has resulted from its use in any case, except the slight smarting that attends its application.

Aug. 1st., 1850.

